# Quorum sensing in streptococci and its peptide-mediated modulation

**DOI:** 10.1042/BSR20260162

**Published:** 2026-06-03

**Authors:** Clay P. Renshaw, Keely M. Rodriguez, Yftah Tal-Gan

**Affiliations:** Department of Chemistry, University of Nevada, Reno, 1664 North Virginia Street, Reno, NV 89557, U.S.A.

**Keywords:** Competence, peptides, Quorum sensing, Streptococci

## Abstract

Quorum sensing (QS) is a density-dependent communication process that enables bacteria to coordinate group behaviors, including competence, biofilm formation, bacteriocin production, and virulence. Early observations of genetic transformation in *Streptococcus pneumoniae* and density-dependent bioluminescence in marine bacteria laid the foundation for the eventual unification of these phenomena under the QS framework. In streptococci, QS is mediated primarily by secreted peptide pheromones that are processed, exported, and sensed through either membrane-associated two-component signal transduction systems or intracellular RRNPP-family regulators. These signaling pathways control tightly regulated and often transient physiological states, most notably competence, through interconnected regulatory circuits such as ComABCDE and ComRS. Increasing evidence indicates that, in some streptococci, these systems do not operate in isolation but instead form integrated and, in some cases, noncanonical networks that respond to environmental and metabolic cues. The chemical accessibility of peptide signals has enabled detailed structure–activity relationship studies, revealing key determinants of receptor activation and specificity. In particular, systematic modification of competence-stimulating peptides (CSPs) and *sigX*-inducing peptides (XIPs) has led to the identification of residues critical for activity and enabled the development of peptide analogs that act as agonists or competitive antagonists of QS pathways. These studies have demonstrated that synthetic peptides can modulate QS-regulated phenotypes, including competence, and in some cases attenuate infection *in vivo*. Collectively, these advances establish streptococcal QS as a chemically tractable system and highlight peptide-based modulation as a strategy for probing and influencing bacterial communication.

## Discovery of quorum sensing

**Figure 1:**
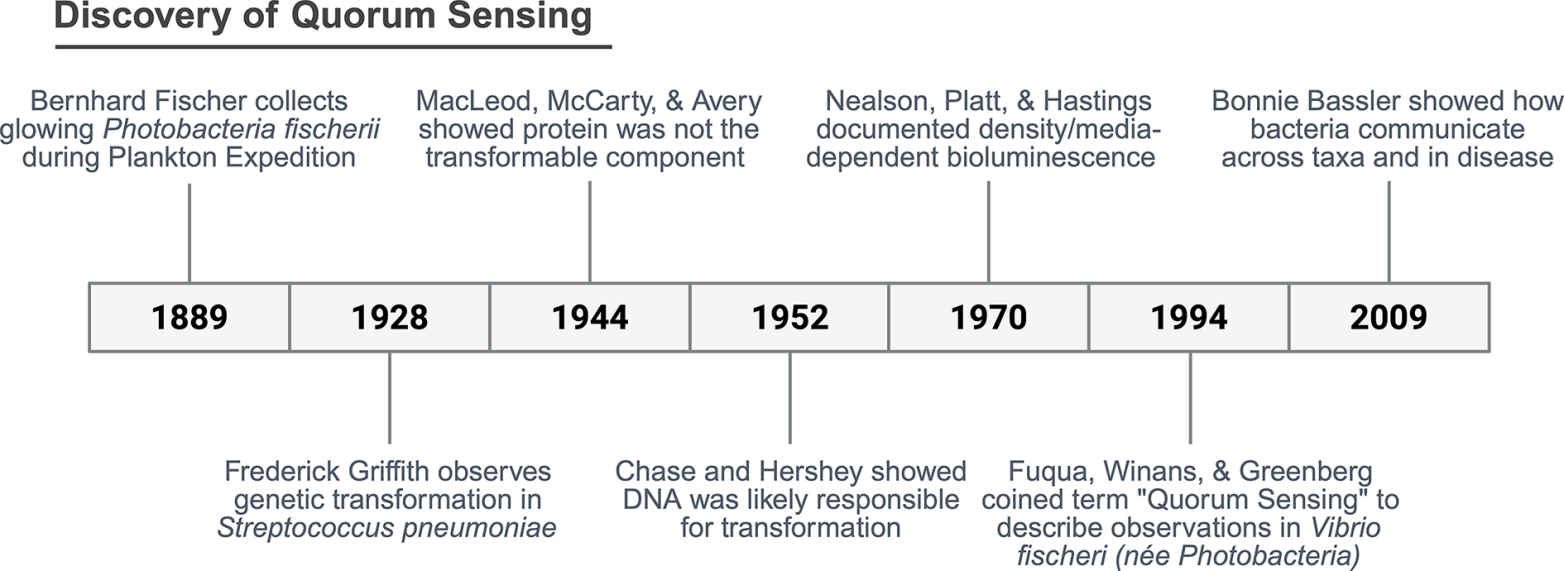
The History of Bacterial QS Discovery Timeline highlighting key discoveries in bacterial QS.

The concept of quorum sensing (QS) emerged across a timeline of early observations of bacterial transformation and coordinated group behaviors (Figure 1). In 1923, Frederick Griffith demonstrated that a “transforming principle” could transfer virulence between *Streptococcus pneumoniae* strains [1], a phenomenon later shown to be mediated by DNA through the work of Oswald Avery, Colin MacLeod, Maclyn McCarty, and subsequently confirmed by Martha Chase [2,3]. As mechanisms of DNA uptake and genetic competence were explored [4,5], parallel studies on bioluminescent bacteria revealed that light production depended on cell density and diffusible “autoinducer” molecules [6–10]. Similar density-dependent behaviors, including bacteriocin production, biofilm formation, and competence, were soon observed across diverse bacterial species, suggesting a unifying principle of coordinated population-level regulation [11–20]. These findings were consolidated under the term “quorum sensing,” popularized in the 1990s by E. Peter Greenberg and colleagues, and later expanded by Bonnie Bassler, whose work demonstrated that such chemical communication can occur both within and between bacterial species [21–27].

## QS and virulence

Once so many cellular processes were found to be controlled by QS, effort was soon made to reveal how these processes could be controlled exogenously [25,28–30]. After all, many of these processes afforded the bacteria increased virulence, making them potentially more dangerous to a host infected with them [28]. QS serves as a population-density-sensing phenomenon that coordinates the timing and magnitude of virulence factor deployment across diverse bacterial pathogens. In Gram-negative bacteria, *N*-acyl homoserine lactone-based circuits typified by LuxI/LuxR homologs regulate a broad array of infection-relevant traits, including biofilm formation, production of extracellular enzymes and toxins, and operation of secretion systems. In *Pseudomonas aeruginosa*, for example, hierarchically organized QS networks (Las, Rhl, and PQS) control elastases, rhamnolipids, phenazines, and biofilm architecture, all of which contribute to tissue damage, immune evasion, and persistence in chronic infections such as those seen in cystic fibrosis [12,31,32]. Similarly, in *Vibrio* species, QS links cell density to the expression of hemolysins, proteases, and type III secretion effectors, enabling a shift from colonization to overt virulence once a sufficient bacterial population has established within the host [33–35].

In Gram-positive bacteria, peptide-mediated QS systems couple the detection of secreted oligopeptides to transcriptional reprogramming of virulence traits. In *Staphylococcus aureus*, the Agr system senses an autoinducing peptide and, at high cell density, up-regulates secreted toxins and degradative enzymes while down-regulating surface adhesins, thereby promoting tissue invasion and dissemination after initial colonization [36–38]. Similarly, the dental pathogen *Streptococcus mutans*, which has been implicated as a major culprit in tooth decay, was found to regulate biofilm formation through peptide-based QS [14,15,17]. *Streptococcus mutans* biofilms formed on the teeth, protect the bacteria inside, and must be physically removed by brushing and flossing. Failure to do so results in the buildup of dental calculus where *S. mutans* remains safe to release lactic acid that eats through the tooth enamel, leading to cavities [39,40]. Oral streptococci were also found to cause diseases such as endocarditis or vasculitis after entering the bloodstream [41,42].

## Streptococcal QS: general features

**Figure 2:**
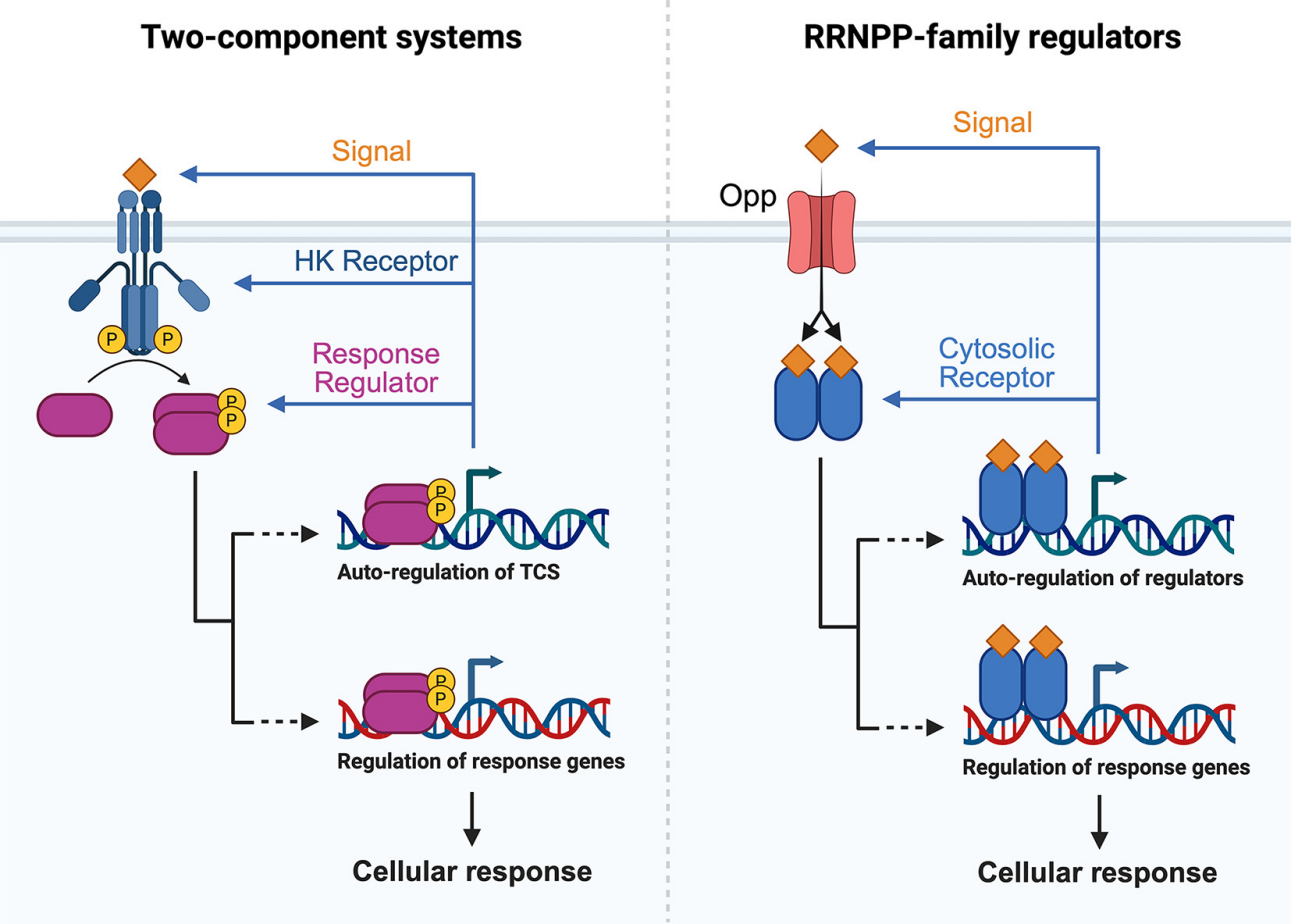
Types of streptococcal QS systems Streptococci generally use one of two types of QS systems: two-component signal transduction systems (left) or RRNPP-family regulatory systems (right). TCSs comprise a HK transmembrane receptor that senses an extracellular QS peptide signal, leading to autophosphorylation and dimerization of the HK receptor and subsequent phosphorylation of a cytosolic response regulator. The phosphorylated response regulator can then dimerize and act as a transcriptional regulator of the entire TCS circuitry as well as downstream response genes responsible for group behaviors. In RRNPP-type systems, the QS peptide signal is imported into the cell via a non-specific Opp, where it can bind its cytosolic RRNPP-family protein receptor, leading to its dimerization and subsequent transcriptional regulatory activity of its own circuitry as well as downstream group behavior genes.

Streptococci encode a rich repertoire of QS systems that use secreted peptide pheromones to coordinate diverse group behaviors, including competence, bacteriocin production, biofilm formation, and virulence [43]. These signaling peptides are typically synthesized as *N*-terminally extended pro-peptides, exported by dedicated active transporters, proteolytically processed to their mature forms, and then sensed extracellularly by receptors on the cell surface or intracellularly by cytosolic transcription factors. The best-characterized peptide-responsive regulators belong to two broad classes: membrane-associated two-component signal transduction systems (TCSs) such as ComDE and BlpRH, and cytosolic RRNPP-family regulators, including ComR and the Rgg subfamily [43–45]. Full-length pro-pheromones (e.g., ComC, ComS, SHPs) are generally processed by exporters with peptidase activity (e.g., ComAB, BlpAB, PptAB), generating short mature pheromones that can accumulate in the local environment [44,46]. These mature peptides either bind the membrane-associated histidine kinase (HK) receptor of the TCS to initiate phosphorylation cascades or are re-imported through the oligopeptide permease (Opp) and directly engage cytosolic receptors (Figure 2). As the population grows, extracellular pheromone concentrations increase until threshold levels are reached, at which point a subpopulation or the entire community undergoes coordinated changes in gene expression, frequently via positive feedback loops that sharply amplify signal output [47,48]. QS outputs in streptococci include regulation of nutrient acquisition, stress tolerance, natural competence and horizontal gene transfer, production of bacteriocins and fratricins, and biofilm architecture, thereby aligning communal behaviors with local environmental conditions and cell density [49–60]. Though TCSs that sense QS peptides extracellularly have been well-studied, genomic surveys have revealed extensive RRNPP repertoires in multiple streptococcal groups, suggesting that intracellular peptide-sensing is a major regulatory logic in this genus [43,61,62].

## Competence as a population-level trait

Bacterial competence—the ability to take up and (in many cases) recombine exogenous DNA—is a tightly regulated, transient physiological state that allows streptococci to acquire new traits such as antibiotic resistance, altered capsule serotypes, and niche-adaptation factors [63–69]. In many streptococci, QS-controlled competence regulons comprise early genes (e.g., *comAB*, *comCDE*, *comX/sigX*) and late genes encoding DNA uptake and recombination machinery (e.g., *comEA*, *comEC*, *dprA*, *recA*) as well as fratricide effectors that lyse non-competent siblings [15,48,60,70–72].

Competence development is typically synchronized with specific growth phases and environmental cues, such that induction occurs only when the population is sufficiently dense and metabolically poised to benefit from DNA uptake [55,73]. Competence tends to peak in early exponential phase and is sharply delimited in time, due to downstream negative feedback and proteolytic shutdown mechanisms [74–76]. This temporal control ensures that the energetic cost of assembling DNA uptake machinery and the risk associated with genome remodeling are restricted to windows when neighboring cells (and hence potential DNA donors) are abundant, and nutrients and stressors favor transformation-linked traits. As previously stated, in addition to DNA uptake, QS-dependent competence regulons also tend to regulate genes that facilitate host colonization and niche-dominance phenotypes, including biofilm formation, stress tolerance, and bacteriocin production, blurring the distinction between “competence” and more general community-level behaviors [49–60].

## ComABCDE competence regulon

**Figure 3:**
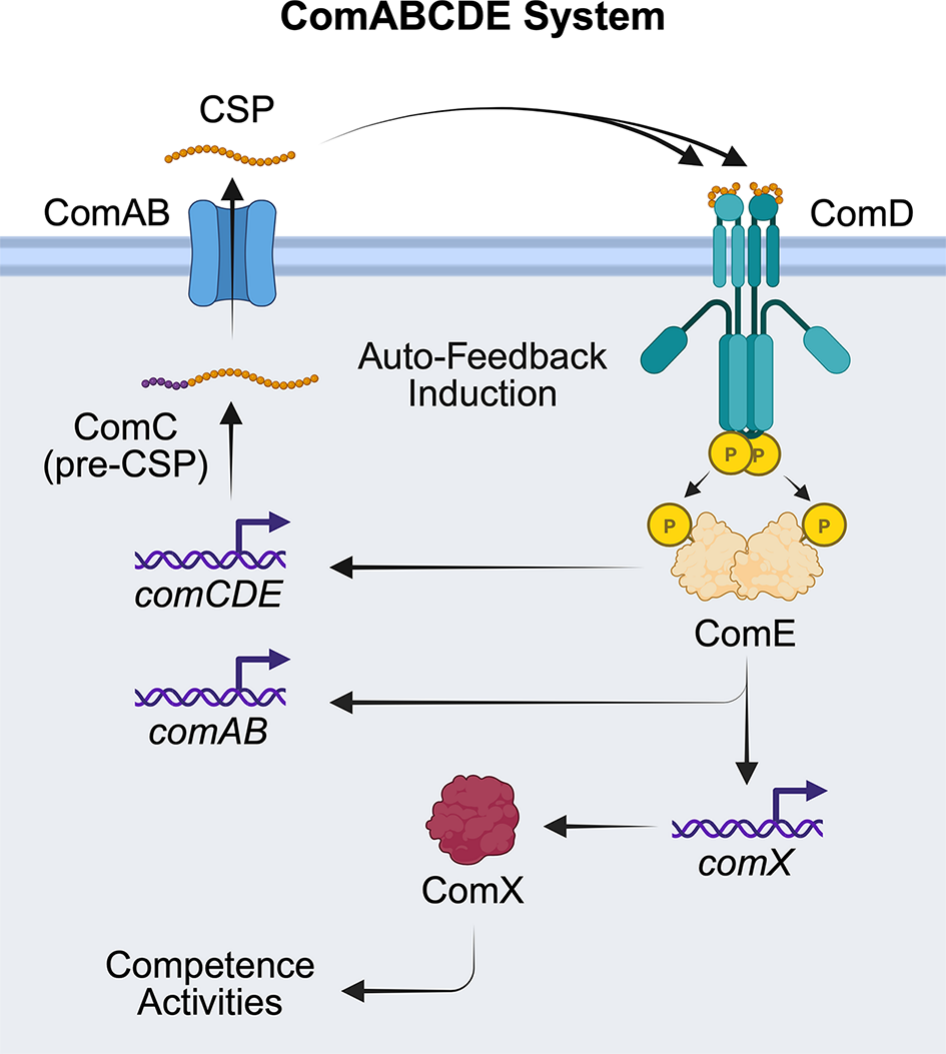
The streptococcal ComABCDE system The ComABCDE system, or “competence regulon,” uses the ComDE two-component system to sense the CSP QS signal and induce auto-feedback expression of the ComABCDE regulon, as well as regulate expression of the alternative sigma factor, ComX, which regulates downstream competence activities. Specifically, after processing and export of ComC by ComAB, the mature CSP accumulates in the environment to bind and activate the ComD receptor. Upon activation, ComD activates ComE via phosphorylation. ComE up-regulates the *comCDE* and *comAB* operons, as well as *comX*, to activate group phenotypes.

The ComABCDE system—often simply termed the “competence regulon”—is the archetypal QS circuit controlling competence in many Mitis and Anginosus group streptococci, among others (Figure 3). The *comC* gene encodes a pro-peptide with an *N*-terminal leader sequence that signals ComC for export from the cell by the ComAB ABC transporter [77]. The ComC sequence contains a double glycine (GG) motif that is recognized for proteolytic processing via ComAB’s peptidase domain, yielding the mature competence-stimulating peptide (CSP) that accumulates extracellularly [77,78]. In general, ComC pro-peptides are about 40–50 residues in length, with their corresponding CSP ranging around 15–20 residues. Once CSP reaches a threshold concentration, it binds to the cognate TCS HK receptor ComD, promoting receptor dimerization, autophosphorylation, and subsequent phosphorylation of the response regulator ComE [44]. Phosphorylated ComE dimerizes and acts as an activator of early competence genes, including *comAB*, *comCDE*, and *comX/sigX*, thereby creating a powerful positive feedback loop that rapidly synchronizes the population’s transition into competence [44,63]. SigX (ComX) is a Sigma-70 family alternative sigma factor that directs RNA polymerase to late competence promoters, driving expression of genes essential for DNA binding, uptake, processing, and recombination, as well as (in *S. pneumoniae*) fratricide factors such as CbpD and bacteriolytic enzymes that lyse non-competent siblings and release DNA [60,64,67,72,79]. This coupling of competence and fratricide allows competent cells to access a rich source of homologous DNA within the same ecological niche, enhancing the efficiency of horizontal gene transfer and facilitating rapid adaptation. In *S. pneumoniae*, ComDE cross-talks with the BlpRH TCS that regulates bacteriocin production, and the two HK can share signals or regulatory targets, integrating competence with antimicrobial peptide production. ComDE-like systems (or closely related BlpRH circuits) are present across many streptococcal groups, although careful phylogenetic and synteny analyses have revealed that several loci once annotated as *comDE* are in fact bacteriocin-specific BlpRH systems, underscoring the evolutionary plasticity and functional diversification of these peptide-responsive TCSs [44,54,61,80].

## ComRS intracellular QS system

**Figure 4:**
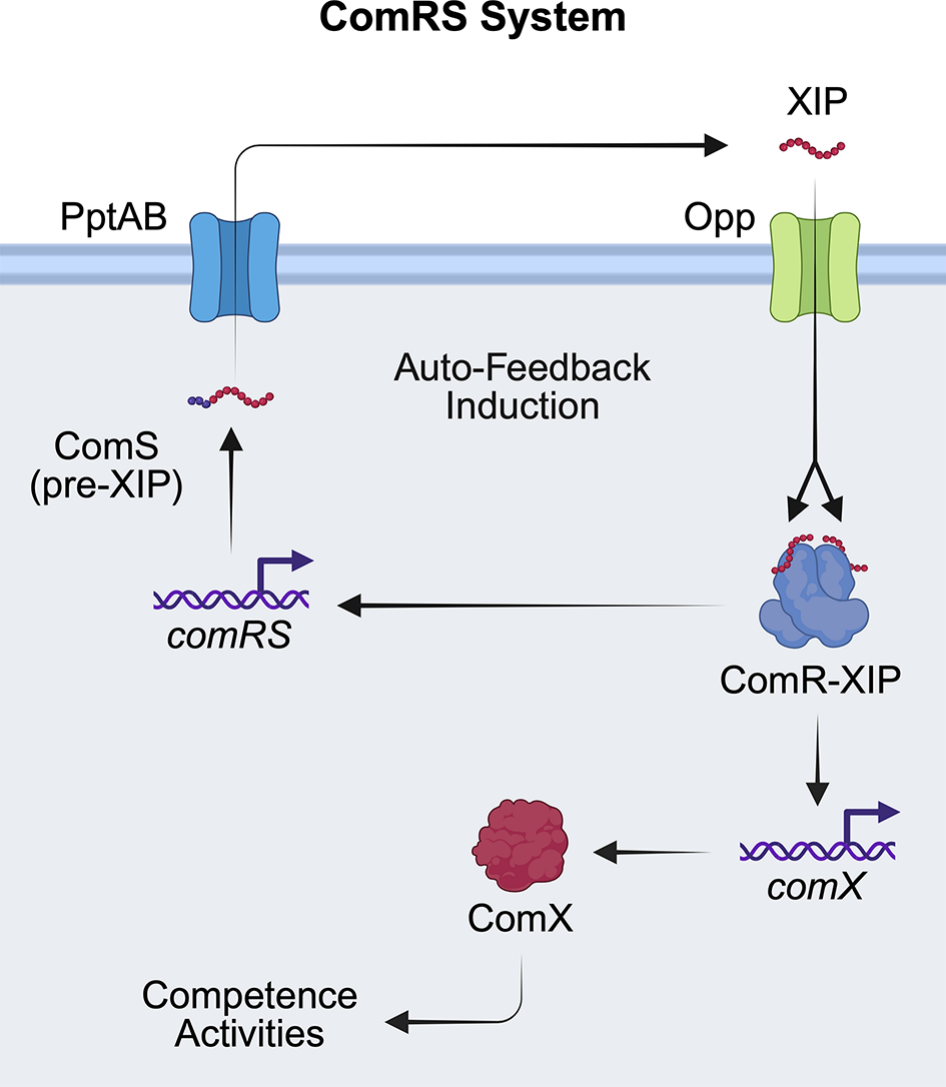
The streptococcal ComRS system The ComRS system uses the RRNPP-type regulator, ComR, to sense the XIP QS signal and induce auto-feedback expression of the ComRS system, as well as regulate expression of the alternative sigma factor, ComX, which regulates downstream competence activities. Specifically, after processing and export of ComS by PptAB, the mature XIP accumulates in the environment and is imported into the cell by the Opp to bind and activate ComR, which acts as a cytosolic receptor. Activated ComR acts as a transcription factor to up-regulate the *comRS* operon and *comX* to activate group phenotypes.

In contrast with the surface-sensing ComABCDE pathway, the ComRS system exemplifies intracellular peptide sensing via RRNPP-family regulators (Figure 4). In general, *comS* encodes a peptide precursor that, after export and proteolytic maturation via the PptAB ABC transporter, yields the short hydrophobic *sigX*-inducing peptide (XIP), which is subsequently re-imported into the cytosol through the Opp and binds the cognate transcription factor, ComR [46,81–84]. ComS lengths can range broadly from around 17–35 residues in length, with the mature XIPs being much smaller, generally 7–8 residues. XIP–ComR complex formation promotes ComR dimerization and binding to promoter regions upstream of *comS* and *comX/sigX*, generating a positive feedback loop that sharply induces ComX activity and thereby competence gene expression [47,85].

Comparative genomic and functional studies indicate that ComRS is the predominant competence-regulating QS system in many streptococcal groups, including Mutans, Salivarius, Pyogenes, Bovis, and Suis lineages, whereas a canonical ComABCDE-SigX axis is largely restricted to Mitis and Anginosus groups [54,59,84,86,87]. Coevolution studies of ComR/ComS pairs across streptococci have revealed tight pheromone-receptor specificity, with distinct ComRS “types” showing strong matching between the XIP motif and its binding pocket in ComR [82–84]. Beyond competence, ComRS-like modules in streptococci can regulate bacteriocins and other fitness traits, and cross-species or even interkingdom stimulation of ComRS circuits has been documented—for example, *Candida albicans* enhances *S. mutans* ComRS-SigX activation in dual-species biofilms [51,57,88].

## Deviations from canonical systems and integrated networks

**Figure 5:**
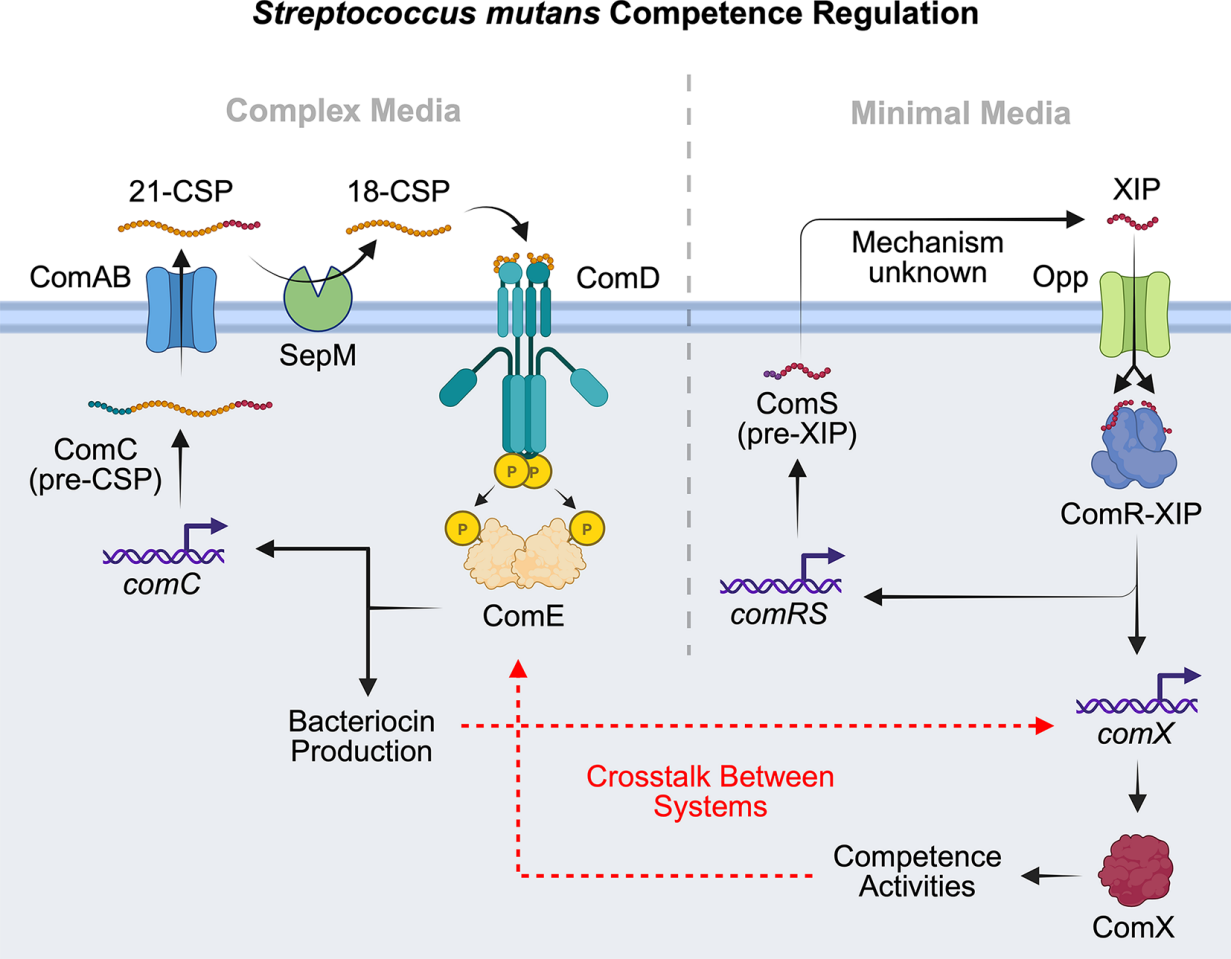
*Streptococcus mutans* competence regulation The bacteriocin production and competence QS pathways in *S. mutans* are controlled by two integrated ComABCDE and ComRS systems, which deviate from the canonical QS circuitry. In the ComABCDE system, ComC is cleaved and exported by ComAB, as usual; however, the produced 21-mer CSP (21-CSP) is further processed by the extracellular protease, SepM, to produce the mature 18-mer CSP (18-CSP). The 18-CSP then activates the ComDE TCS, as usual, but ComE then regulates bacteriocin production in this system instead of competence activities. The ComRS system in *S. mutans* appears to function as usual to regulate competence activities; however, the ComS processing machinery in this system has not yet been identified. The ComABCDE system is mainly stimulated by CSP in complex media, while the ComRS system is mainly stimulated by XIP in minimal media, likely as an ecological adaptation to fast-and-feast cycles in the oral microbiome. Cross-talk between the two systems also occurs when each system is activated (highlighted in red), suggesting a potential dual-QS signal integration network for controlling group behaviors in response to multiple environmental cues.

While many streptococci rely on either ComABCDE or ComRS to regulate competence, a growing number of species exhibit noncanonical or integrated QS architectures. *Streptococcus mutans* is the best-studied example: it encodes both a ComDE-like TCS (historically misannotated but now recognized as part of a BlpRH-family bacteriocin pathway) and a ComRS module, and these networks are interconnected in complex, medium-dependent ways (Figure 5) [54,89–93]. ComABCDE activity in *S. mutans* primarily regulates mutacin bacteriocin production but can also induce competence through regulatory cross-links, whereas ComRS directly controls *comX* expression and late competence genes [88]. In this system, competence induction by exogenous XIP occurs only under chemically defined or minimal media conditions, driving a largely unimodal competence response across the population [91]. In contrast, exogenous CSP addition induces competence only in complex or nutrient-rich media conditions, yielding a bimodal competence response, with only a subpopulation of cells activating ComX [89,91]. This competence signaling relationship between the ComABCDE and ComRS systems in *S. mutans* reflects a complex integration of peptide signaling with nutrient availability, illustrating a potential evolutionary adaptation to the fast-feast cycles of the oral microbiome.

Some streptococci can also deviate from the canonical export and processing machinery. In *S. mutans*, a 21-mer CSP is produced through the ComAB exporter, but this peptide is further processed by the extracellular SepM protease to produce the active 18-CSP [40,94,95]. Additionally, in *Streptococcus gallolyticus subsp. gallolyticus*, a gallocin-stimulating peptide (GSP) structurally related to CSP activates a bacteriocin operon, but the mature pheromone begins three residues downstream of the predicted GG leader, implying non-standard processing rules and unique interactions with the BlpAB transporter [96]. Furthermore, recent work in *Streptococcus sanguinis* revealed a noncanonical ComCDE circuit in which the typical ComAB exporter is absent and pheromone secretion is instead partitioned between two distinct ABC transporters, while the ComC processing machinery has yet to be identified [73].

Beyond competence, numerous Rgg/SHP systems in *Streptococcus pyogenes* and related species modulate biofilm development, virulence factor expression, and metabolic adaptation through short hydrophobic peptides (SHPs) that follow the same export, maturation, import, and Rgg-binding paradigm as the ComRS system [59,97]. These RRNPP systems are often present in multiple paralogous copies within a single genome, and cross-reactivity among their pheromones can create intricate intra- and inter-strain signaling networks that further diversify QS outputs [43,62,98]. Additional peptide circuits are now being discovered—for example, a recently described MutRS system in *S. mutans* controls lantibiotic mutacin production through a dedicated pheromone and displays extensive inter- and intraspecies cross-talk, further expanding the QS vocabulary in oral streptococci [61]. Combinatorial architectures, in which multiple peptide circuits intersect, are emerging as a recurring theme across bacteria [31,99]. Together, these examples highlight that streptococcal QS is best viewed as an interconnected signaling landscape rather than a single linear pathway.

## Peptide modulators of streptococcal QS biology

The advent and maturation of solid-phase peptide synthesis have enabled precise chemical interrogation of streptococcal QS systems, allowing exogenous peptides to be used as probes and candidate therapeutics [79,100–103]. Accessibility to efficient peptide synthesis in the past few decades has made it possible to chemically recreate and systematically manipulate signaling peptides like CSP and XIP, converting QS from an observational field to an engineerable system. This also brought along abilities to incorporate non-proteogenic amino acids, including D-amino acids and unnatural amino acids, for the purpose of modulating QS processes in pathogenic bacteria. Systematic structure-activity relationship (SAR) studies of streptococcal CSPs have identified key determinants of ComD binding and activation, including the essential role of an *N*-terminal acidic residue (Asp/Glu) for receptor activation and a conserved Arg at position 3 necessary for receptor engagement, in many species (Table 1) [40,104–112]. Substituting the *N*-terminal Asp/Glu with Ala often yields dominant-negative CSP analogs that competitively inhibit native CSP binding and block competence development [104–107,112,113].

**Table 1:** Activity data for streptococcal QS peptides and select analogs.

Species	QS Peptide	Sequence	EC_50_/IC_50_* (nM)	Observed phenotypes modulated	Ref.
*S. constellatus*	CSP	DS**R**IRMGFDFSKLFGK	5.2	Competence	[104]
	CSP-D1A	ASRIRMGFDFSKLFGK	120*	Competence	[104]
	CSP-I4A	DSRARMGFDFSKLFGK	0.089	Competence	[104]
*S. cristatus*	CSP	DL**R**NIFLKIKFKKK	52.6	Competence, H_2_O_2_ production, interspecies inhibition	[105]
	CSP-D1A	ALRNIFLKIKFKKK	>1000*	Competence, H_2_O_2_ production	[105]
*S. gallolyticus*	GSP 21-mer	DFLIVGPFDWLKKNHKPTKHA	2.96	Bacteriocin production	[96]
	GSP-des-D1-G6	PFDWLKKNHKPTKHA	3.19	NA	[96]
*S. gordonii*	CSP	DI**R**HRINNSIWRDIFLKRK	282	H_2_O_2_ production, interspecies inhibition	[106]
	CSP-D1A	AIRHRINNSIWRDIFLKRK	>1000*	NA	[106]
	CSP-d1	dIRHRINNSIWRDIFLKRK	>1000*	NA	[106]
	CSP-desD1	IRHRINNSIWRDIFLKRK	985*	NA	[106]
*S. mitis*	CSP-2	EI**R**QTHNIFFNFFKRR	148	Competence	[114]
	CSP-2-E1A	AIRQTHNIFFNFFKRR	456*	NA	[114]
	CSP-2-E1Af10r16	AIRQTHNIFfNFFKRr	87.3*	Competence, biofilm formation	[114]
*S. mutans*	18-CSP	SGSLSTFFRLFNRSFTQA	6.2	Interspecies inhibition	[40,110]
	P23 CSP Analog	GSLSAFFRAFNAAFTAA	0.8	Interspecies inhibition	[110]
	XIP	GLDWWSL	440	Competence	[115]
	XIP-G1AD3AW4AS6A	ALAAWAL	10,000*	Competence	[115]
*S. oligofermentans*	CSP	DS**R**NIFLKIKFKKK	65.7	Competence, H_2_O_2_ production	[107]
	CSP-D1A	ASRNIFLKIKFKKK	957*	NA	[107]
	CSP-D1AK14A	ASRNIFLKIKFKKA	537*	NA	[107]
	CSP-cyc(K2E8)	D(KRNIFLE)IKFKKK	9.29	NA	[116]
	CSP-D1AK14A-cyc(Dap2D8)	A(DapRNIFLD)IKFKKA	355*	NA	[116]
*S. pneumoniae*	CSP1	EM**R**LSKFFRDFILQRKK	10.3	Pneumolysin production	[112,117]
	CSP1-E1A	AMRLSKFFRDFILQRKK	85.7*	NA	[112]
	CSP2	EM**R**ISRIILDFLFLRKK	50.7	Pneumolysin production	[112,117]
	CSP2-d10	EMRISRIILdFLFLRKK	2.86	NA	[112]
	CSP2-E1Ad10	AMRISRIILdFLFLRKK	56.5*	NA	[112]
	CSP1-cyc(Dap6E10)	EMRLS(DapFFRE)FILQRKK	12.2 (ComD1), 31.4 (ComD2)	NA	[117]
	CSP1-E1A-cyc(Dap6E10)	AMRLS(DapFFRE)FILQRKK	75.8* (ComD1), 182* (ComD2)	Pneumolysin production, lung infection and mortality	[117]
*S. sanguinis*	CSP	DLRGVPNPWGWIFGR	14.4	NA	[73]
*S. sinensis*	CSP	DS**R**RLNFGGFIKFFGK	8.72	NA	[118]
	CSP-D1A	ASRRLNFGGFIKFFGK	293*	NA	[118]

In these SAR studies, bolded R3 positions in the native QS peptide sequences were found to be critical for receptor activity, highlighting the conserved functional nature of this position. “Ref.” = Reference.

Several CSP analogs, including linear CSP-E1A variants and more recent cyclic peptides, have demonstrated potent inhibition of the pneumococcal competence regulon at low nanomolar concentrations and have shown efficacy in mouse models of acute pneumonia and bacteremia, reducing bacterial loads and improving survival [108,117,119–122]. Importantly, some of these analogs act as pan-group inhibitors capable of intercepting ComD1 and ComD2 signaling, and cross-group modulation can be further enhanced by exploiting CSPs from closely related Mitis group species, such as *S. mitis*, as scaffolds [108,117,121]. Beyond anti-virulence potential, these studies provide detailed insights into receptor-ligand recognition and the sequence tolerance of CSPs, informing broader design rules for peptide-based QS modulators.

Analogous efforts have targeted ComRS circuits. In *S. mutans*, SAR analyses of XIP have mapped residues critical for ComR recognition and activation, leading to the identification of potent XIP-derived QS modulators [115]. Because ComRS systems are widespread and often more specific than ComABCDE-like pathways, XIP-based inhibitors hold promise for species-selective modulation of competence and associated phenotypes in oral streptococci.

Collectively, these synthetic peptide studies underscore several recurrent themes. In CSPs, *N*-terminal acidic residues and specific conserved positions are often crucial for receptor activation, and substitutions at the *N*-termini frequently produce competitive antagonists. Cyclization or backbone constraint can enhance proteolytic stability and *in vivo* efficacy. Exploiting natural cross-reactivity among QS systems (e.g., between *S. mitis* and *S. pneumoniae* CSPs) can yield broad-spectrum agonists or antagonists with translational potential. As computational peptide design tools (e.g., BindCraft, BoltzGen, RFdiffusion, LigandMPNN, PepMLM, PepTune) and protein-ligand modeling tools (e.g., AlphaFold, OpenFold, Chai-1, RoseTTAFold) continue to improve in accessibility and accuracy, they are poised to accelerate the rational design of next-generation QS inhibitors and biased agonists in streptococci [123–132].

## The future of streptococcal QS research

Future work on streptococcal QS is likely to move along several converging axes: systems-level dissection of network architecture, ecological and polymicrobial context, and translational exploitation of peptide signaling. At the systems level, single-cell and time-resolved approaches have already revealed that competence induction in *S. mutans* can be heterogeneous and excitable, with QS circuits integrating metabolic cues, stress responses, and additional peptide inputs to generate bimodal or localized behaviors rather than simple homogeneous on/off switches [88,92]. Expanding such analyses across species and growth environments will be crucial for understanding how ComABCDE, ComRS, Rgg/SHP, MutRS, and other TCSs or RRNPP systems are wired together within a given strain and how evolutionary rewiring reshapes the mapping from signal to phenotype.

From an ecological perspective, streptococcal QS must be interpreted within the complex, polymicrobial communities these organisms inhabit, such as dental plaque, the nasopharynx, and the gut. Interactions with neighboring bacteria and even fungi can modulate or hijack QS circuits, as exemplified by *C. albicans*-driven activation of the *S. mutans* ComRS pathway and the influence of Mitis group CSPs on pneumococcal competence [57,108]. Large-scale comparative genomics of RRNPP repertoires and TCS families across streptococci is beginning to reveal lineage-specific expansions and biases such as the overrepresentation and synchronous activation of SHP/Rgg systems in *S. thermophilus* [62], which likely reflect adaptation to distinct ecological niches and community structures.

Translationally, peptide-based QS modulators offer an attractive anti-virulence strategy that disarms pathogens by preventing competence, horizontal gene transfer, and expression of key virulence factors, rather than relying on bactericidal activity that strongly selects for resistance. The success of CSP analogs in attenuating pneumococcal infection *in vivo* provides a compelling proof-of-concept, but further development will require exploring pharmacokinetics, delivery routes, and specificity to minimize off-target effects on commensal streptococci. Additionally, combination treatments of QS inhibitors with antibiotics may yield synergistic outcomes, as has recently been observed, for example, with the addition of the QS inhibitor hamamelitannin increasing *S. aureus* susceptibility to vancomycin [133,134]. In parallel, deciphering how QS controls bacteriocin production, fratricide, and biofilm formation in commensal streptococci could enable probiotic or microbiome-engineering strategies that leverage QS-tuned commensals to suppress pathogenic colonization.

As new QS pathways, novel pheromones, and unexpected cross-talk mechanisms continue to be uncovered, streptococci will remain a fertile model for understanding how peptide-based communication shapes community behavior, as well as for translating that understanding into therapies that target non-essential communication pathways.

## Abbreviations

CSPs: competence-stimulating peptides
GSP: gallocin-stimulating peptide
HK: histidine kinase
Opp: oligopeptide permease
QS: Quorum sensing
SHPs: short hydrophobic peptides
XIPs: *sigX*-inducing peptides
SAR: structure-activity relationship
TCSs: two-component signal transduction systems
